# Use of a Remote Car Starter in Relation to Smog and Climate Change Perceptions: A Population Survey in Québec (Canada)

**DOI:** 10.3390/ijerph6020694

**Published:** 2009-02-16

**Authors:** Diane Bélanger, Pierre Gosselin, Pierre Valois, Stéphane Germain, Belkacem Abdous

**Affiliations:** 1 Centre de recherche du CHUQ, 2875 boulevard Laurier, Édifice Delta 2, Québec, QC, Canada G1V2M2; E-Mail: diane.belanger@crchul.ulaval.ca; 2 Institut national de santé publique du Québec, Ouranos and Université Laval, 945 Avenue Wolfe, Québec, QC, Canada G1V 5B3; 3 Faculté des Sciences de l’éducation, Université Laval, Québec, QC, Canada G1V0A6; E-Mail: pierre.valois@fse.ulaval.ca; 4 Département de mathématiques et de statistique, Université Laval, Québec, QC, Canada G1K7P4; E-Mail: stephane.germain@gmail.com; 5 Centre de recherche du CHUQ, 2875 boulevard Laurier, Édifice Delta 2, Québec, QC, Canada G1V2M2; E-Mail: belkacem.abdous@crchul.ulaval.ca

**Keywords:** Air pollution, car idling, climate change, environment and public health, health-related behavior

## Abstract

Remote car starters encourage motorists to warm up their vehicles by idling the motor – thus increasing atmospheric pollutants, including several greenhouse gas (GHG) with impacts on public health. This study about climate change (CC) adaptation and mitigation actions examined perceptions on air pollution and climate change and individual characteristics associated with the use of a remote car starter. A telephone survey (n = 2,570; response rate: 70%) of adults living in Québec (Canada) measured the respondents’ beliefs and current behaviours regarding CC. Approximately 32.9% (daily car users) and 27.4% (occasional users) reported using a remote car starter during winter. The odds of the use of a remote car starter was higher in the less densely populated central (OR: 1.5) and peripheral regions (OR: 2.7) compared to the urban centers (ex. Montreal). The odds was also higher in population with a mother tongue other than English or French (OR: 2.6) and francophones than anglophones (OR: 2.1), women than men (OR: 1.5), daily drivers than occasional ones (OR: 1.2), and respondents who at least sometimes consulted temperature/humidity reports than those who consulted them less often (OR: 1.5). In multivariate analysis, the perception of living in a region susceptible to winter smog, being aware of smog warnings, or the belief in the human contribution to CC did not significantly influence the use of a remote car starter. The use of remote car starters encourages idling which produces increased atmospheric pollution and GHG production and it should be more efficiently and vigorously managed by various activities. A five-minute daily reduction in idling is equivalent to reducing the total car emissions by 1.8%. This would constitute a “no-regrets” approach to CC as it can simultaneously reduce GHG, air pollution and their health impacts.

## 1. Introduction

Remote car starters are electronic devices allowing the automatic start of a car from a distance of up to 1.5 kilometres. They can be used to warm up the car in winter time or to cool it down in summer time. The duration of such warming or cooling varies with outside temperature, often taking several minutes per episode, with the associated combustion related pollution and greenhouse gas (GHG) production. In Quebec, one car in ten was equipped with a remote car starter in 2006, while other regions in Canada had much lower rates, hovering between 1 and 4% [1]. These Canadian estimates were associated with high absolute numbers, as in 2005, Québec had a total of about 4.2 million light vehicles (which includes cars, station wagons, vans, sport utility vehicles and pick-ups) and other regions, approximately 13.8 million [2]. This means between half a million and one million light vehicles owners can use their remote starters in Canada for idling their vehicles. While no data seems available on remote starter use globally, it represents an annual $250-million market in the United States of America, which means that more than a million such devices get installed on cars every year [3].

However, no detailed data is systematically collected by Canadian authorities to document the use of this technology. Nevertheless, Natural Resources Canada reports that people with remote starters tend to start their vehicles long before there are ready to drive, throughout the year [4], most commonly to warm them up in winter [5], instead of less polluting alternatives such as using cloth seat covers, dressing appropriately or plugging in the car in the morning (or overnight) to warm up the coolant and/or engine oil or to feed AC powered car heaters.

In fact, idling an engine to warm it rather than driving for approximately 30 seconds after a cold-start is not only unnecessary according to experts (because a vehicle’s engine and other parts warm up faster when the vehicle is moving), but also an habit which produces more pollution than if the engine were shut off and restarted [6]. Consequently if every driver of a light vehicle in Canada reduced by only five minutes daily the time that his vehicle idled, it would prevent more than 1.4 million tonnes of CO_2_ being emitted into the atmosphere [7], which is equivalent to a reduction of 320,000 automobiles travelling for an entire year, or 1.8 % of the total vehicle fleet. Besides this reduction is that of emissions of fine particulates and other transport-related atmospheric pollutants (e.g. sulphur dioxide, nitrous oxide, carbon monoxide) other than CO_2_, of which some also have a greenhouse effect (e.g. nitrous oxide), as well as the reduction of health impacts related to air pollution, particularly in young children, the elderly, people with respiratory problems (e.g., asthmatics) or people with a heart condition [8–14]. Those population groups are also among the most vulnerable to health impacts from climate change [15].

Clearly, remote car starters encourage motorists to warm up their vehicles by idling the motor – a polluting habit with impacts on public health which becomes even more problematic with the increasing supply and demand for this type of technology in Canada [1]. The aim of this study was to examine diverse perceptions and individual characteristics associated with use of a remote car starter in winter (among other climate change related behaviors) through a survey carried out in 2005 in southern Québec (Canada) in the context of a research program on some climate change (CC) adaptation and mitigation strategies [16].

## 2. Methods

### 2.1. Study Population and Sample

**Figure 1.**
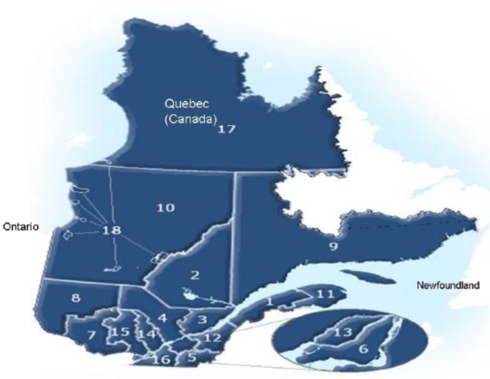
Administrative Health and Social Services Regions, Québec (Canada). Source: MSSS, Service des Infocentres, 2006. Legend: Eastern part of southern Québec: 1 (Bas-Saint-Laurent), 9 (Côte-Nord) and 11 (Gaspésie Îles-de-la-Madeleine); Northern part of southern Québec: 2 (Saguenay Lac-Saint-Jean) and 8 (Abitibi-Témiscamingue); Québec city region: 3 (Québec) and 12 (Chaudière-Appalaches); Central Québec: 4 (Mauricie Centre-du-Québec); North of Montréal: 7 (Outaouais), 14 (Lanaudière) and 15 (Laurentides); South of Montréal: 5 (Estrie) and 16 (Montérégie); Montréal and Laval: 6 (Montréal), 13 (Laval)

The study population consisted of adults aged 18 years or older from the southern part of the Province of Québec south of the 49th parallel, namely all the health regions presented in Figure 1, with the exception of sub-arctic regions 10, 17 and 18.

The sample was stratified by the health region of residence, and post-stratified by gender (in order to take into account the greater difficulty in reaching men [17]). Due to operational and budgetary constraints, we used random household sampling instead of a within-household sampling. The respondents were contacted by a polling firm from random digit dialing of published residential telephone numbers (confidential numbers were not used due to ethical considerations). The study obtained ethical approval from Laval University’s *Comité d’éthique de la recherche avec des êtres humains*. The sample size was calculated using 2001 survey data [18], for a 95% confidence level and a precision level of 1.5%, for a 4-point Likert-type scale including 6 items [19]. From the initial sample (n = 4,000), 2,570 completed the questionnaire (Table 1), for a response rate of 70%. The percentage of respondents and non-respondents were similar across health regions of Québec (p = 0.4).

### 2.2. Data Collection Method

The polling firm collected individual responses by telephone (average duration: 20 minutes), seven days a week, from 9:30 a.m. to 9:30 p.m., using a computer system that allowed the order of the questions (essentially closed) to be randomly redistributed. More precisely, collection (from 15-09-2005 to 25-10-2005) allowed information to be gathered on socio-demographic characteristics, health status, dwelling, region of residence, the use of an automobile and a remote starter during the whole winter, consultation of weather and smog reports, as well as on perceptions and beliefs relating to climate change and the behaviors adopted during a period of intense cold. The questionnaire was developed according to the following six steps: 1) identifying the important issues to consider in the exploratory interviews [20] based on the literature on health and climate change; 2) conducting 21 face-to-face pilot interviews (average duration: two hours), mainly to verify the understanding of some terms, identify the items to be retained as well as the sensitive issues to be excluded; 3) development of an initial version of the questionnaire; 4) conducting telephone interviews with 61 people aged 18 years or older (on average, four people per health region studied) to validate the clarity and precision of the questions, to comment on the questionnaire and to shorten it; 5) validation of the content of the questionnaire (French and English versions) by five experts working in the field of health and climate change in Canada; 6) conducting of a qualitative pretest (n=50) (two versions of the questionnaire) by the polling firm, at the start of each data collection.

### 2.3. Analyses

The collected information was calibration weighted for the respondent’s age and language, on the basis of 2001 census data [21]. Coefficients of variation (CV) – or relative standard deviation [22] – were calculated (CV < 15%: sufficiently precise estimates; CV between 15% and 25%: acceptable precision, estimates to be carefully interpreted; CV>25%: low precision, estimates to be interpreted with circumspection) [23]. The percentages totals for a given variable may not be exactly 100%, due to rounding to the closest decimal (to simplify the presentation, percentages below 2% for missing data have not been reported). The analyses took into account the sample scheme stratified according to the health regions [24,25]. Using a remote car starter in winter was related to the independent variables with the help of the Rao-Scott likelihood ratio chi-square test, which is a design-adjusted version of the Pearson chi-square test. The multivariate analyses were done using a logistic regression model with a stepwise method (significance level required to include in the model: 0.2; to stay in the model: 0.1). The c index (area under the ROC curve; expected value = 0.5 to 1.0) was used as an indicator of the discriminant capacity of the final multivariate statistical model [26]. Finally, the presence of collinearity between the independent variables was checked (VIF > 10; condition > 30) [27].

## 3. Results

### 3.1. Characteristics of the Respondents

Women accounted for slightly more than half of the sample, as well as did people 35 to 64 years of age (Table 2). At least two participants out of three lived in a house and spoke only French (Table 2), except in Montréal and Laval (Table 3). More than half of the respondents (56.8%) used a car every day, and 27.0%, less than once a day (never: 16.2%). In the first group, 32.9% used a remote car starter in winter and in the second group, 27.4%.

### 3.2. Factors Associated with the Use of a Remote Car Starter in Winter

The prevalence of use of a remote car starter in winter was higher for women than for men, as well as for francophones, or people with a mother tongue other than English or French (called allophones in Canada), compared to anglophones (Table 4). Higher percentages of respondents using a remote car starter at least occasionally during the winter were observed for those individuals living in a house, in particular in the peripheral regions of southern Québec than in the more urban environments (Table 4), such as Montréal (Table 3). Similarly, higher percentages of respondents using this technology were observed for those individuals who considered their region of residence to be less prone to winter smog, ice storms or cold waves. Using a remote car starter in winter was more frequent among respondents who consulted the meteorological information (temperature, intense cold warning, and humidity rate) in the media, than for the other participants consulting it rarely or never.

However, no statistical differences were observed between the users and non users of a remote car starter for the adaptation of clothing behaviours to the weather report (e.g. clothing warmer than usual if cold warning) or for the observance of other preventive advice (e.g. remote car starter not to be used if smog warning).

Finally, using a remote car starter in winter was most frequent among respondents who did not believe at all in the anthropogenic contribution to climate change over the last 50 years, compared to participants who believed in it. Among 31 multivariate sub-models (2^5^−1), the most discriminant model included five of the variables associated with the use of a remote car starter (c index: 0.6239). This model seemed to differentiate users from non-users, on the basis of: (1) living in the peripheral regions; (2) respondents’ sex; (3) first language learned at home; (4) consultation of weather reports (temperature or humidity rate) in the media; (5) using a car every day.

More specifically (Table 5), compared to the respondents living in the adjacent cities of Montréal and Laval, the odds of using a remote car starter was 1.5 times higher for the participants living in other central regions of southern Québec (e.g. regions 3–5 in Figure 1) and 2.7 times for those in the most peripheral regions (e.g. regions 2 or 9 in Figure 1). The odds were also 1.2 higher for the participants driving a car every day than for occasional drivers; 1.5 times higher for women than men; respectively 2.1 and 2.6 times higher for francophones and allophones, than for anglophones. Finally, the odds of using a remote car starter was 1.5 times higher for the respondents who consulted (at least sometimes) weather reports (temperature or humidity rate) than for respondents who rarely or never consulted it.

## 4. Discussion

This population survey on beliefs and adaptations about climate change, including the use of remote car starters, did not intend to measure the determinants of that behaviour, the impact of such use on car idling, the levels of air pollutants, nor the impact of pollutants on the health of the population.

The survey is broadly representative of the Québec adult population for the variables presented in Table 2. It also brings to light that among the 83.8% of respondents used a car – which is very close to the percentage of 81% of Québec households reported in the Canadian Households and Environment Survey [21] – approximately one-third used a remote car starter in winter. Furthermore, using a remote car starter in winter was not influenced by smog warnings. From a public health standpoint, these results are of concern for several reasons.

Firstly, vehicle exhaust from idling (related in part to the use of remote car starters) contribute to air pollution and climate change [28]. Even in densely populated city centers where many people use public transport (such as Montréal in this survey), outdoor air quality can be severely affected by vehicle idling, at the local (ex. around schools) or community level [7–11]. Concerning greenhouse gases (GHG), between 1990 and 2005, Canada’s transport sector has increased its share of emissions by 33% and is responsible for the equivalent of 32% of the total observed GHG emissions growth [29].

Air pollution – of which primary sources include vehicle exhaust – is known to cause a variety of adverse health effects, ranging from minor illnesses to emergency room visits, hospital admissions and premature death [30–32]. For example, for the Québec regions where complete data on atmospheric pollution was available in 2002 (covering roughly half of the population of Québec, or 3,6 million people), exposure to fine particulates, ozone and nitrous oxides was associated (using prudent assumptions) to 1,974 premature deaths, 414 emergency room visits for respiratory problems, 38 emergency room visits for cardiac problems, and to 246,705 days with asthma symptoms [33]. Currently, specific and periodic monitoring of vehicle idling (including the part related to remote car starter use) and its impact on atmospheric pollutants and GHG emissions does not exist in Canada.

Secondly, as found in this study, using a remote car starter does not seem to be influenced by the smog warnings and preventative recommendations issued by Environment Canada through the media and other initiatives. Incentive and voluntary programs such as Info-Smog and the Auto$mart Program have been in existence respectively since 1994 [34] and 1998 [6], apparently with little behavioural impact [35]. Moreover, there were only 61 Canadian municipal and community initiatives against motor vehicle idling in 2005, among the more than 3,000 Canadian municipalities. Only 26 of these initiatives were considered as regulatory, either by governing idling specifically, or by including clauses against it in other existing regulations [5,36]. The remaining 35 initiatives were of the voluntary-approach type to behavioural change, such as public education campaigns or incentive programs.

Thirdly, in this study, the prevalence of use of a remote car starter was higher for women than for men, allophones and francophones than for anglophones, in more rural than urban regions. Possible explanations for such variations could include higher perceived intensity of cold for women [37] or allophone (ex. immigrants from tropical countries) [38], distinct clothing habits (ex. clothing consisting of fabrics providing less efficient retention of the heat given off by their bodies, such as rayon [39]), or even the fact of living in a region characterized by colder winters (ex. regions 2 and 8, Figure 1) [40]). Given that drivers living in peripheral regions are more dependent on their cars for daily trips [41], they are susceptible to using remote starters more often. However, more research is needed to understand why some people have a greater propensity to use a remote car starter, because the significant differences were not very strong (c index: 0.62), indicating the potential contribution of other types of factors like driver differences in the trip chaining behaviour related, for instance, to children care or other family chores [42], social factors, attitudes and beliefs [43,44]), such as the belief on the contribution of anthropogenic causes to climate change in the last fifty years. The availability of remote car starters also seems to incite motorists to develop and maintain the easier habit to warm up their vehicles with a remote starter by idling the engine instead of adopting less polluting strategies, such as dressing appropriately. Such studies would help policy maker’s better target education campaigns supporting behaviour modification programs.

Consequently, in Canada and other similar Nordic regions (ex. USA or Northern Europe), it would be appropriate to implement long-term comprehensive national programs to reduce all types of light vehicles idling, including actions against the use of remote car starters, to reduce pollutant and GHG emissions at the source. It is indeed likely that remote car starters are also used in summer to cool down cars by the now widely available air-conditioners, thus increasing their contribution to air pollution. Such programs could simultaneously merge feasible adaptation and mitigation measures of the “no-regrets” type (which are measures with climatic and non-climatic benefits), including both voluntary and regulatory tools, to deal effectively with environmental problems. These risks management measures could include:

1. specific and periodic monitoring of the idling phenomenon;
2. legislative and regulatory framework updates, including sunset dates to phase out remote starters (for users, vehicle builders and installation shops);
3. dynamic technological improvement of new vehicles (ex. devices that automatically cut off an engine after 10 seconds of immobilization while on park or with braking, already available in hybrid vehicles [45]);
4. simultaneous actions such as public education, incentive or regulatory campaigns aimed at individual and collective behaviours, taking cultural differences into account and based on results of health behaviour research; and,
5. evaluation and monitoring of all of these approaches.

Our results, however, show that even with good intentions and voluntary incentive programs, behavioural change remains difficult. Emphasis on the elimination of remote car starters and accelerated introduction of automatic shut-off devices are likely to be more effective in our opinion, given the only marginal impact of even well-publicized smog advisories in Canada [35].

This population survey on beliefs and current behaviours about climate change presents some limitations, however. As mentioned earlier, it did not intend to measure the impact of using remote car starters on the levels of air pollutants (including several greenhouse gases) associated with idling motor of vehicles, nor the impact of related pollutants on human health. This has limited the scope of questioning on the specific topic of use of remote car starters, its determinants and impacts.

Furthermore, for financial and operational reasons, only the household was random sampled. It is possible that respondents interviewed by the polling firm were most inclined to participate to the study, that if they were be randomly chosen among all the persons composed an household, even though the reverse remains another possibility [46–48]. In the same way, the full socio-demographic profile of the non-respondents remains unknown and cannot be compared to the profile of the respondents.

Although our response rate (70%) for this telephone survey is considered very good in the polling industry, many potentially eligible individuals did not agree to participate in the study. Volunteer bias could have possibly influenced the study results, but the percentage of respondents and non-respondents were similar across health regions of Québec (p = 0.4). This poll also excludes automatically unpublished numbers, inhabitants not speaking either French or English and homeless people. On the statistical side, the index C for the retained model is not very high at 0.62; this might be explained by the absence of the above-mentioned factors related to the determinants of behaviour. The addition of such psychosocial variables could probably improve the model performance, moreover by using another dataset for the validation of our results.

## 5. Conclusions

Implementation of a long-term national program on controlled and reduced vehicle idling, including phasing out remote car starters and accelerated phasing in of automatic shut-off devices, as part of “no-regrets” adaptation and mitigation measures to climate change could contribute to attenuating climate change, to reducing air pollution and increasing health and quality of life of the population. Research on the cultural and psychosocial determinants of such practices could help focus future intervention programs.

## Figures and Tables

**Table 1.** Sample description.

A) Initial sample		4,000	

B) Not-valid numbers		129	
	No service		89
	Non-residential		13
	Fax/modem/cellular/paget		27

C) Numbers excluded from sample		140	
	Foreign language		12
	Completed quota (for stratified sampling)		70
	Non qualified (ex. disease, age under 18 years)		54
	Bad quality of communication		4
Effective sample (A minus B+C)		3,731	
Non completed interview		1,161	
	Refusal		646
	No response		155
	Answering machine		129
	Occupied		3
	Incomplete		11
	Not interviewed because data collection ended before the date of the appointment made with the polling firm		217

D) Completed interviews		2,570	
Response rate (C+D/A-B)		70.0%	

**Table 2.** Sociodemographic characteristics of the respondents: percentages corrected for stratified sampling, and coefficients of variation.

Variables		% 1	CV 2

Gender	Women	51.6	0.02
	Men	48.3	0.02

Age	18 to 34 years	29.1	0.03
	35 to 64 years	54.6	0.02
	65 years or more	16.2	0.05

First language learned at home	French only	81.0	0.01
	English only	6.1	0.09
	Language other than French or English	10.1	0.15
	English or French plus another language	2.9	0.08

Status of activities (last 12 months)	Employed	67.0	0.02
	Unemployed	8.4	0.07
	Student	3.4	0.15
	Retired	21.8	0.04

Income (before tax/from all sources/last 12 months)	Less than $ 15 000	9.3	0.07
	Between $ 15 000 and $ 29 999	17.2	0.05
	Between $ 30 000 and $ 44 999	17.8	0.05
	Between $ 45 000 and $ 59 999	14.1	0.05
	$ 60 000 and or more	26.2	0.03
	Undisclosed 3	15.2	0.05

Lived alone	Yes	18.2	0.04
	No	81.8	0.01

Region of residence	Eastern Québec	5.7	0.02
	Northern part of southern Québec	5.9	0.02
	Québec City region	14.6	0.01
	Centre of the province	6.4	0.02
	South of Montréal	21.1	0.01
	North of Montréal	15.7	0.01
	Montréal and Laval	30.8	0.01

Type of dwelling	House	64.9	0.01
	Apartment: ≤ 4 storeys	31.1	0.03
	Apartment: ≥ 5 storeys	3.9	0.11

1 %: percentages. The total percentages for a given variable may not be exactly 100%, due to rounding to the closest decimal. To simplify the presentation, percentages below 2% for missing data have not been reported.

2 CV: coefficients of variation. CV < 15%: sufficiently precise estimates; CV between 15% and 25%: acceptable precision, estimates to be carefully interpreted; CV > 25%: low precision, estimates to be interpreted with circumspection.

3 These participants, compared to those who disclosed their income strata, were more often women, individuals at least 65 years of age, and retired people.

**Table 3.** Some characteristics of the respondents by region of residence: percentages corrected for stratified sampling.

Variables	Region of residence						
	Eastern Québec	Northern part of southern Québec	Centre of the province	Québec City region	South of Montréal	North of Montréal	Montréal and Laval
Type of dwelling:							
•house	87.4 % 1	78.8 %	76.0 %	67.2 %	73.8 %	85.0 %	38.4 %
•apartment	12.6 %	21.2 %	24.0 %	32.9 %	26.3 %	15.0 %	61.6 %
First language learned at home:							
•French only	96.0 %	95.0 %	96.2 %	93.4 %	86.1 %	85.3 %	60.8 %
•other then French only	4.0 %	5.0 %	3.8 %	6.6 %	13.9 %	14.7 %	39.2 %
Region of residence perceived as prone to cold waves:							
•average or a lot	71.6 %	81.4 %	78.4 %	81.5 %	81.5 %	80.7 %	81.9 %
•not much or not at all	28.5 %	18.7 %	21.6 %	18.5 %	18.6 %	19.3 %	18.1 %
Region of residence perceived as prone to ice storms:							
•average or a lot	17.8 %	9.4 %	20.4 %	20.5 %	29.1 %	24.7 %	46.9 %
•not much or not at all	82.2 %	90.7 %	79.6 %	79.6 %	70.9 %	75.3 %	53.1 %
Region of residence perceived as prone to winter smog:							
•average or a lot	42.6 %	35.9 %	56.0 %	42.2 %	69.6 %	64.3 %	63.0 %
•not much or not at all	57.4 %	64.1 %	44.0 %	57.8 %	30.4 %	35.7 %	37.0 %
Using a car:							
•never	9.1 %	8.0 %	7.8 %	14.6 %	10.7 %	8.4 %	29.4 %
•less than once a day	33.7 %	25.3 %	30.4 %	25.4 %	28.4 %	26.5 %	25.6 %
•every day	57.2 %	66.7 %	61.8 %	60.1 %	61.0 %	65.1 %	45.0 %
Using a remote car starter in winter:							
•without car	9.1 %	8.0 %	7.8 %	14.6 %	10.7 %	8.4 %	29.4 %
•yes	34.3 %	49.0 %	26.2 %	34.0 %	25.3 %	31.2 %	16.8 %
•no	56.6 %	43.1 %	59.2 %	58.2 %	64.1 %	60.4 %	53.8 %

1 The total percentages for a given variable may not be exactly 100%, due to rounding to the closest decimal. To simplify the presentation, percentages below 2% for missing data have not been reported.

**Table 4.** Use of a remote car starter in winter in southern Québec for various respondents characteristics: percentages corrected for stratified sampling, and p value.

Variables	Remote car starter		
	yes	no	p value 1

*Sociodemographic characteristics*			
Gender:			< 0.0001
•women	35.3 % 2	64.7 %	
•men	27.1 %	72.9 %	

First language learned at home:			0.0008
•English only	16.2 %	83.84 %	
•French only	32.6 %	67.4 %	
•Other language	31.5 %	68.6 %	

Status as parent:			0.1376
•no children	28.1 %	71.9 %	
•adult children only	33.4 %	66.6 %	
•at least one minor child	31.6 %	68.5 %	

Cohabitation:			0.1097
•lives with other people (related or not)	31.9 %	68.1 %	
•lives alone	27.4 %	72.6 %	

*Dwelling and region of residence*			
Type of dwelling:			0.0574
•house	32.5 %	67.5 %	
•apartment, building≤4 storeys	28.9 %	71.1 %	
•apartment, building≥5 storeys	19.6 %	80.4 %	

Region lived in:			< 0.0001
•Eastern Québec	37.7 %	62.3 %	
•Northern part of southern Québec	53.2 %	46.8 %	
•Central Québec	36.9 %	63.1 %	
•Québec City region	30.6 %	69.4 %	
•North of Montréal	34.0 %	66.0 %	
•South of Montréal	28.3 %	71.7 %	
•Montréal and Laval	23.8 %	76.3 %	

Region of residence perceived as prone to ice storms:			0.0386
•a lot	29.4 %	70.6 %	
•average	28.5 %	71.5 %	
•not much	35.3 %	64.7 %	
•not at all	33.8 %	66.2 %	

Region of residence perceived as prone to winter smog:			0.0140
•a lot	28.4 %	71.6 %	
•average	26.1 %	73.9 %	
•not much	31.5 %	68.5 %	
•not at all	34.4 %	65.6 %	

Region of residence perceived as prone to cold waves:			0.0444
•a lot	27.5 %	72.5 %	
•average	34.2 %	65.8 %	
•not much	30.5 %	69.5 %	
•not at all	30.1 %	69.9 %	

Using a car:			0.0130
•less than once a day	27.5 %	72.5 %	
•every day	32.9 %	67.1 %	

*Meteorological reports in the media*			
Consultation of temperature:			0.0168
•often or always	32.5 %	67.5 %	
•sometimes	29.1 %	70.9 %	
•rarely or never	22.8 %	77.3 %	

Consultation of intense cold warning:			0.0923
•often or always	32.6 %	67.4 %	
•sometimes	29.7 %	70.3 %	
•rarely or never	26.6 %	73.4 %	

Consultation of humidity rate:			0.0225
•often or always	34.6 %	65.4 %	
•sometimes	28.0 %	72.0 %	
•rarely or never	29.2 %	70.8 %	

*Outings despite the intense cold wave*			
For shopping:			0.0058
•often or always	29.2 %	80.8 %	
•occasionally	30.3 %	69.7 %	
•rarely or never	38.2 %	61.8 %	

For intense physical activities outdoors (e.g. running):			0.0054
•often or always	27.3 %	82.7 %	
•occasionally	30.7 %	69.3 %	
•rarely or never	35.1 %	64.9 %	

More layers than usual:			< 0.0001
•always	26.1 %	73.9 %	
•often	29.4 %	70.6 %	
•occasionally	39.7 %	60.3 %	
•rarely	40.2 %	59.8 %	
•never	36.7 %	63.3 %	

Head covering:			0.0991
•always	29.5 %	70.5 %	
•often	28.7 %	71.3 %	
•occasionally	36.5 %	63.5 %	
•rarely	39.3 %	60.7 %	
•never	36.6 %	63.4 %	

Scarf:			0.0773
•always	30.0 %	70.0 %	
•often	28.9 %	71.1 %	
•occasionally	39.8 %	60.2 %	
•rarely	41.6 %	58.4 %	
•never	30.0 %	70.0 %	

Belief on the contribution of anthropogenic causes to climate change in the last fifty years:			0.0554
•a lot	29.4 %	70.6 %	
•average	32.0 %	68.0 %	
•not much	33.0 %	67.0 %	
•not at all	40.9 %	59.1 %	

^1^ Use of remote car starters was related to the independent variables using the Rao-Scott likelihood ratio chi-square test, which is a design-adjusted version of the Pearson chi-square test. Non significant variables included: Age, activity status in last 12 months, income from all sources in last 12 months, perceived health status, having at least one chronic disease diagnosed by a physician for last six months, requires a technical aid for outdoor trips, requires accompaniment (animal or person) for outdoor trips, perceived influence of extreme meteorological conditions on health, consultation of smog warnings, adaptation of clothing behaviour to weather conditions, observance of preventive advice for smog or extreme meteorological conditions, clothing warmer than usual in some conditions.

^2^ The total percentages for a given variable may not be exactly 100%, due to rounding to the closest decimal. To simplify the presentation, percentages below 2% for missing data have not been reported.

**Table 5.** Indicators differentiating respondents using a remote car starter in winter from non-users: multivariate analysis corrected for stratified sampling.

	Remote car starter in winter		
	OR^1^	CI_95%_^1^	p value^2^

Region of residence:			< 0.0001
•Montréal and Laval	reference group		
•central regions other than Montréal and Laval	1.5	1.2 ; 1.8	
•most peripheral regions of southern Québec	2.7	1.8 ; 3.5	

Gender:			0.0002
•men	reference group		
•women	1.5	1.2 ; 1.8	

First language learned at home:			0.0087
•English only or other language in addition to French/English	reference group		
•French only	2.2	1.3 ; 3.5	
•Allophone (other than French and English)	2.6	1.3 ; 5.0	

Using a car:			0.0426
•not every day	reference group		
•every day	1.2	1.1 ; 1.5	

Consultation weather reports (temperature or humidity rate) in the media:			0.0434
•rarely/never	reference group		
•at least sometimes	1.5	1.0 ; 2.2	

^1^ OR: odds ratio; CI_95%_: 95% confidence interval. The odds ratios presented in this table indicate the capacity of a variable to discriminate the participants using a car starter in winter from those that do not. For example, the odds of using a car starter was 1.5 times was higher for women than for men. The value of c index was 0.62, which is low. No collinearity between the independent variables was observed.

2 The p value associated with the Wald test was obtained using logistic regression.

## References

[1] Print Measurement Bureau. Table 43. Automotive reports 2007 http://www.pmb.ca/public/e/product_data/reports_online_gateway.shtml? (accessible by subscription).

[2] Natural Resources Canada. Canadian vehicle survey 2005: summary report 200560 http://oee.nrcan.gc.ca/Publications/statistics/cvs05/pdf/cvs05.pdf (accessed 2008).

[3] Gilroy A (2006). TWICE: This Week in Consumer Electronics.

[4] Natural Resources Canada. Idling and Climate Change Go Hand in HandNatural Resources Canada, 2008 http://www.oee.nrcan.gc.ca/transportation/idling/why-idle.cfm?attr=16 (accessed January 29, 2009).

[5] Natural Resources Canada. Report: Driver Behaviour Affecting Fuel ConsumptionOffice of Energy Efficiency, 1998 http://www.oee.nrcan.gc.ca/transportation/idling/issues/why-idling-problem.cfm?attr=16 (accessed December 23, 2008).

[6] Penney J Clean Air Partnership Cracking down on idling: a primer for Canadian municipalities on developing and enforcing idling control by-lawsOffice of Energy Efficiency, 2005 http://oee.nrcan.gc.ca/communities-government/transportation/municipal-communities/reports/index.cfm?attr=8 (accessed April 2008).

[7] Natural Resources Canada Ecoenergy for personal vehicles Natural Resources Canada 2008 http://oee.nrcan.gc.ca/transportation/idling/impact.cfm?attr=28 (accessed April 2008).

[8] Stefani D Mohapatra A Public health implications of traffic density and vehicle idling on air quality 2008 http://www.oee.nrcan.gc.ca/transportation/business/documents/Idling-reports/health-backgrounder.cfm?attr=16 (accessed April 2008).

[9] Chen Y, Craig L, Krewski D (2008). Air quality risk assessment and management. J. Toxicol. Environ. Health A.

[10] World Health Organisation (Europe). Air quality Guidelines Global Update 2005 World Health Organisation (Europe) Copenhagen, Denmark 2006 496 http://www.euro.who.int/Document/E90038.pdf(accessed on January 29, 2009).

[11] Friedman MS, Powell KE, Hutwagner L, Graham LM, Teague WG (2001). Impact of changes in transportation and commuting behaviors during the 1996 Summer Olympic Games in Atlanta on air quality and childhood asthma. JAMA.

[12] Dominici F, Peng RD, Bell ML, Pham L, McDermott A, Zeger SL (2006). Fine particulate air pollution and hospital admission for cardiovascular and respiratory diseases. JAMA.

[13] Environment Canada Statistics Canada Health Canada Canadian environmental sustainability indicators 2006 51 http://www.statcan.ca/english/freepub/16-251-XIE/16-251-XIE2006000.pdf(accessed April 2008).

[14] Goldberg MS, Burnett RT, Bailar JC, Tamblyn R, Ernst P, Flegel K, Brook J, Bonvalot Y, Singh R, Valois MF, Vincent R (2001). Identification of persons with cardiorespiratory conditions who are at risk of dying from the acute effects of ambient air particles. Environ. Health Perspect.

[15] Gosselin P, Bélanger D, Doyon B, Séguin J (2008). Health Impacts of climate change in Quebec, Chap. 6. Human Health in a Changing Climate: A Canadian Assessment of Vulnerabilities and Adaptive Capacity.

[16] Bélanger D Gosselin P Valois P Abdous B Vagues de froid au Québec méridional: adaptations actuelles et suggestions d'adaptations futures 2006 183 http://www.inspq.qc.ca/pdf/publications/537-VaguesFroid_Quebec.pdf (accessed April 2008).

[17] Alavi A Beaumont J-F Evaluation and adjustment for non-response in the Canadian Labour Force Survey Statistics Canada Ottawa, Canada 2003 http://www.statcan.ca/bsolc/english/bsolc?catno=11-522-X20030017598 (accessed April 2008).

[18] Institut de la statistique du Québec Recensement de la population 2001 : le Québec 2001 http://www.stat.gouv.qc.ca/regions/lequebec/quebec_index.htm#population (accessed April 2008).

[19] Thompson SK (1987). Sample size for estimating multinomial proportions. Amer. Statist.

[20] Presser S, Rothqeb JM, Couper MP, Lessler JT, Martin E, Singer E (2004). Methods for testing and evaluating survey questionnaires.

[21] Statistics Canada Households and the Environment 2006Statistics Canada, Canada,2007 65 http://www.statcan.ca/english/freepub/11-526-XIE/11-526-XIE2007001.pdf (accessed April 2008).

[22] Scherrer B (1984). Biostatistique.

[23] Institut de la statistique du Québec Enquête sociale et de santé 1998 Institut de la statistique du Québec Québec, Canada 2001 642 http://www.stat.gouv.qc.ca/publications/sante/pdf/e_soc98v2-2.pdf (accessed April 2008).

[24] Sautory O (2005). Colloque francophone sur les sondages, Rapport de colloque. Atelier sur les procédures SAS d'échantillonnage et d'analyse de données d'enquête.

[25] R Development Core Team R: A language and environment for statistical computing R Development Core Team Wien Austria 2006 http://www.R-project.org (accessed April 2008).

[26] Hosmer DW, Lemeshow S (1989). Applied logistic regression.

[27] Kleinbaum DG, Kupper LL, Muller KE (1988). Applied regression analysis and other multivariate methods.

[28] Health Canada Air pollution, climate change and your health Health Canada Canada 2002 http://www.hc-sc.gc.ca/ewh-semt/pubs/air/pollution_e.html (accessed April 2008).

[29] Environment Canada National inventory report, 1990–2005: greenhouse gas sources and sinks in Canada Environment Canada Fredericton, New Brunswick, Canada 2008 http://www.ec.gc.ca/pdb/ghg/inventory_report/2005_report/s3_2_eng.cfm#s3_2 (accessed April 2008).

[30] Ontario Medical Association (2005). The illness costs of air pollution: 2005 2026 health and economic damage estimates.

[31] Chiotti Q Lavender B. Ontario Chap. 6 From impacts to adaptation: Canada in a changing climate 2007 Lemmen D Warren F Bush E Lacroix J Natural Resources Canada Ottawa, Canada 2008 227 274 http://adaptation.nrcan.gc.ca/assess/2007/pdf/front_e.pdf (accessed April 2008).

[32] Judek S Jessiman B Stieb D Vet R Estimated Number of Excess Deaths in Canada due to Air PollutionAir Health Effects Division, Health Canada, 2004 http://www.hc-sc.gc.ca/ahc-asc/media/nr-cp/2005/2005_32bk2-eng.php (accessed October 2005).

[33] Bouchard M Smargiassi A Estimation des impacts sanitaires de la pollution atmosphérique au Québec: essai d'utilisation du Air Quality Benefits Assessment Tool (AQBAT) 2008 30 http://www.inspq.qc.ca/publications/notice.asp?E=p&NumPublication=817 (accessed 2008).

[34] Environment Canada (2003). Winter info-smog program for the Southern Quebec regions.

[35] Stieb DM, Paola J, Neuman K (1996). Do smog advisories work? Results of an evaluation of the Canadian Smog Advisory Program. Can. J. Public Health.

[36] Lura Consulting The carrot, the stick, and the combo: a recipe for reducing vehicle idling in Canadian communities Natural Resources Canada Ottawa, Canada 2008 http://www.oee.nrcan.gc.ca/communities-government/transportation/municipal-communities/reports/carrot-stick-combo/index.cfm?attr=28 (accessed April 2008).

[37] Harju EL (2002). Cold and warmth perception mapped for age, gender, and body area. Somatosens. Motor Res.

[38] Beaudreau P Besancenot J-P Caserio-Schönemann C Cohen JC Dejour-Salamanca D Empereur-Bissonnet D Ernie Y Ilef D Laaidi K Ledrans M Le Tertre L Medina S Pascal M Froid et santé : éléments de synthèse bibliographique et perspectives : rapport d'investigation Institute De Veille Sanitaire Saint-Maurice, cedex, France 2008 48 http://www.invs.sante.fr/publications/2004/froid_et_sante/rapport_froid_et_sante.pdf (accessed April 2008).

[39] Joyal F Enfin l'hiver Sclérodermie Québec Sainte-Julie, Québec, Canada 2007 15 http://www.ssvq.org/pdf/info_pt_Raynaud.pdf (accessed June 2006).

[40] Cardinal F (2007). Le mythe du Québec vert.

[41] Turcotte M Dependence on cars in urban neighbourhoods: life in metropolitan areas Canadian Social Trends 2008 21 32 http://www.statcan.ca/english/freepub/11-008-XIE/2008001/article/10503-en.htm (accessed April 2008).

[42] Baldwin G Fagan S Trip chaining while driving, comparing men's and women's behaviour 2008 http://www.statcan.ca/english/freepub/16-002-XIE/2007003/10455-en.htm (accessed 2008).

[43] Core Groupe Behavioral determinants inter-working group meeting: at the Academy for Educational Development 9 11 2003 Washington, DC, USA 2003 44 http://www.coregroup.org/working_groups/Determinants_meeting_rpt0903.pdf (accessed April 2008).

[44] Fishbein M, Triandis HC, Kanfer FH, Becker M, Middlestadt SE, Baum AS, Revenson TA, Singer JE (2000). Factors influencing behavior and behavior change. Handbook of health psychology.

[45] Natural Resources Canada Saving fuel: tips on driving and maintenance Office of Energy Efficiency Ottawa, Canada 2008 http://fleetsmart.nrcan.gc.ca/idling-reduction-toolkit/section14.cfm?attr=16 (accessed 2008).

[46] Schöbi N Joye D À la recherche du bon échantillon : comparaison des résultats entre méthode des quotas et aléatoire 2001 21 http://www.sidos.ch/publications/f_ns_dj_sampling.pdf (accessed February 2006).

[47] Brogan DJ, Denniston MM, Liff JM, Flagg EW, Coates RJ, Brinton LA (2001). Comparison of telephone sampling and area sampling: response rates and within-household coverage. Am. J. Epidemiol.

[48] Glaser SL, Stearns CB (2002). Reliability of random digit dialing calls to enumerate an adult female population. Am. J. Epidemiol.

